# Multidisciplinary management of giant cervicothoracic cutaneous squamous cell carcinoma

**DOI:** 10.1016/j.ijscr.2019.07.068

**Published:** 2019-08-01

**Authors:** P.A. López, M. Pedraza, A. Moreno, O. García, R. Buitrago, G. Mogollon, L. Fory, H. Conrado

**Affiliations:** a Department of General Surgery, Bosque University, Colombia; b Department of Soft Tissue and Breast, National Institute of Cancer, Bogotá, Colombia; c Department of Thorax Surgery, National Institute of Cancer, Bogotá, Colombia; d Department of Head and Neck Surgery, National Institute of Cancer, Bogotá, Colombia; e National Institute of Cancer, Bogotá, Colombia; f Bosque University, Colombia; g Department of General Surgery, Universidad Militar Central, Bogotá, Colombia; h South Colombian University, Colombia

**Keywords:** Skin cancer, Squamous cell carcinoma, Cutaneous, Invasion, Metastasis

## Abstract

**Introduction:**

Cutaneus squamous cell carcinoma (SCC) is the second most common type of nonmelanoma skin cancer. SCC is a malignant neoplasm of the skin characterized by aberrant proliferation of keratinocytes.

**Presentation case:**

We report multidisciplinary surgical management of a 36-year-old male who presented with a huge cutaneous protruding tumor of the cervicothoracic wall. Clinically, he presented with a growing mass on the anterior cervicothoracic wall 3 years ago. Microscopic examination revealed SCC. He underwent complete excision of the lesion. The patient neglected attending our oncological department for receiving chemotherapy. Today, one year after surgery, he is alive without evidence of disease recurrence.

**Discussion:**

SCC treatment depends on location of the lesion, involvement of neighboring structures, functional level of the patient, and the patient’s acceptance of the proposed management strategy.

**Conclusion:**

We conclude that SCC can grow to a huge size if left untreated. Surgery by a multidisciplinary surgical team is the primary mode of treatment, followed by chemotherapy if necessary.

## Highlights

• Non treated squamous cell carcinoma could present as an uncontrollable and substantial disaurement neoplasm.
• Squamous cell carcinoma that are diagnosed early and successfully treated by surgical excision, has a better forecast.
• When a patient present with a gigant squamous cell carcinoma always requires a multidisciplinary team.

## 1 Introduction

Squamous cell carcinoma (SCC) is a malignant tumor characterized by keratinocytes arising in the epidermis, with histological evidence of dermal invasion [1]. It is the second most common type of skin cancer, and its incidence has been increasing steadily [2,3]. Numerous genetic alterations have been described in SCC sub-types, although the molecular mechanisms contributing to tumor initiation and progression are still being studied [4]. Although most cutaneous SCCs are diagnosed early and successfully treated by surgical excision, in a small proportion of cases, especially if neglected, they may show uncontrollable growth and substantial disfigurement and may possess features associated with a high likelihood of recurrence, metastasis, and death [5,6]. Such cases involving giant cutaneous SCCs (maximum diameter >5 cm) can be very difficult to treat and can present with recurrence and/or metastasis despite aggressive excision. Here, we present a case involving a huge cutaneous tumor of the cervicothoracic wall that was excised with optimal clinical results. This work has been reported in line with the SCARE criteria [16].

## 2 Presentation of case

**Fig. 1:**
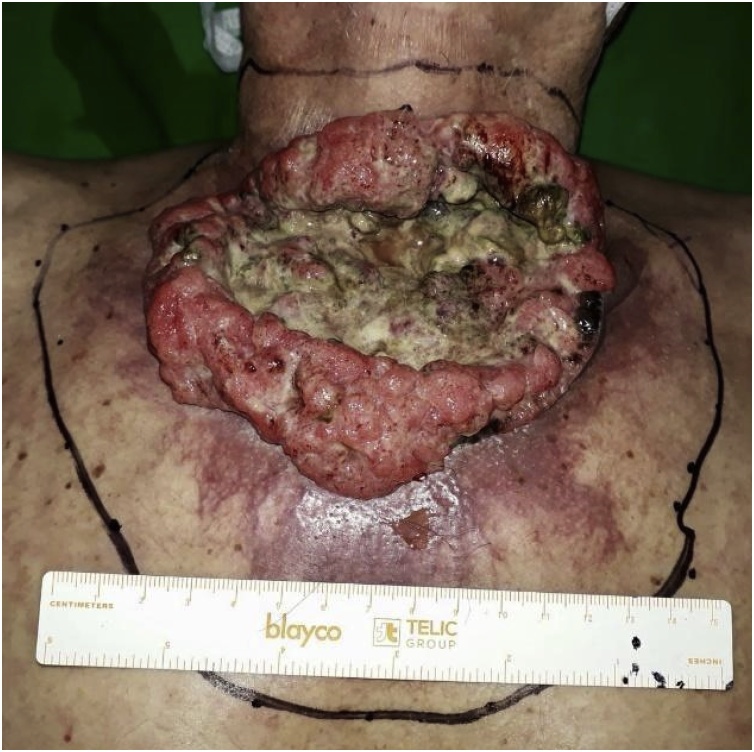
Cervicothoracic giant cutaneous squamous cell carcinoma.

**Fig. 2:**
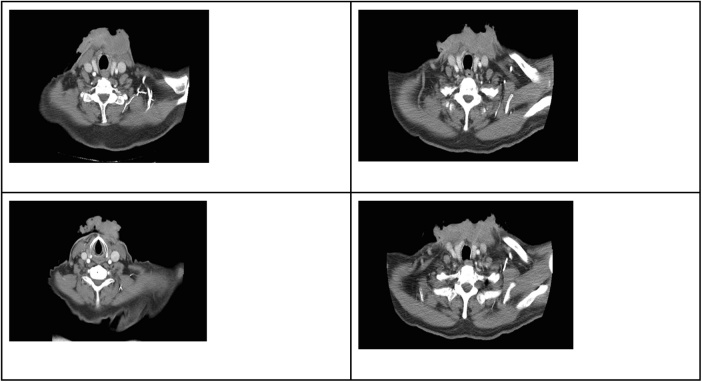
Contrast computed tomography scan of the thorax, showing the appearance of an irregular mass.

A 36-year-old male presented with a 3-year history of a growing mass on the anterior cervicothoracic wall. Biopsy of the lesion revealed moderately differentiated epithelial proliferation with focal keratinization consistent with cutaneous SCC. The mass was a protruding, ulcerated, multilobular, mostly necrotic, foul-smelling, cauliflower-like, firm tumor with hyperkeratotic zones, measuring 11 × 9.5 cm in size that had developed over a large region of erythematous skin (Fig. 1, Fig. 2). He had multiple adenomegalies in the left inframandibular region and right armpit. Complete blood count and biochemistry were normal, and total-body computed tomography (CT) showed that the tumor had not infiltrated deeply into the musculoskeletal layers of the thoracic wall.

His medical history included a personality disorder for which he took medication intermittently because of poor compliance and social support. No lymph node or distant metastases were noted. He consented to surgical reconstruction, and the tumor was excised completely with a 2–3 cm clear margin around it. The tumor appeared to have infiltrated the subcutaneous tissue, and thus, a 4-cm margin of subcutaneous tissue was excised with the tumor. Following this, wide tumor excision of the surrounding skin could not be approximated, and the surrounding skin was left to heal slowly with re-epithelialization.

Neck CT showed a lesion infiltrating the inferior margin of the sternocleidomastoid muscle and prethyroid muscles in the deep plane. The tumor was connected to the anterior margin of the isthmus and the upper margin of the left thyroid lobe, extending to the level of the thyroid cartilage. At its anterior margin, the tumor showed signs of ulceration and had a diameter of approximately 135 × 43 × 65 mm. Thoracic CT showed an irregular-appearing, ulcerated mass in the anterior margin of the base of the neck, without any apparent signs indicating compromise of the visceral space. The mass was in contact with the anterior margin of the sternal handle (Fig. 2). Needle aspiration biopsy of the axillary node was performed and was negative for malignancy. Based on the patient’s condition, oncologists considered her to be a candidate for surgical resection for improving overall survival.

**Fig. 3:**
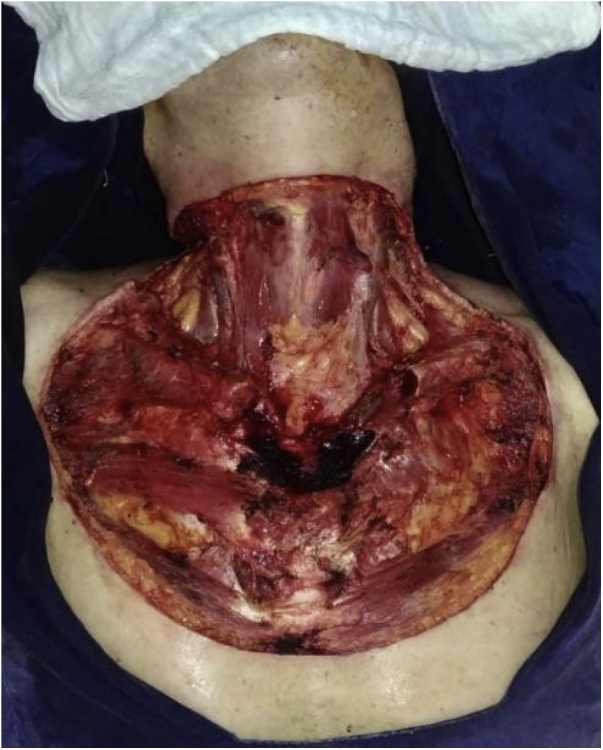
Wide local resection of the neck tumor.

**Fig. 4:**
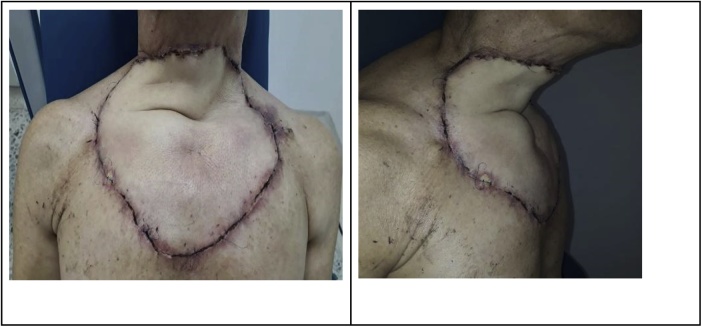
Post-operative results 1 month after surgery.

Multidisciplinary management was performed by surgery of the breast and soft tissues, head and neck, thorax, plastic and reconstructive microsurgery deparments; intraoperative findings showed an ulcerated, necrotic, and stinking tumor lesion measuring 15 × 12 cm in the cervical region with compromise of subcutaneous cellular tissues, prethyroid muscles, inner third of the left clavicle and anterior wall of sternal notch. Then, we decided to perform wide local resection of the neck tumor before margins marking (prethyroid muscles, sternocleidomastoid plus bilateral low parietal decompression plus dissection of blood vessels) neck plus bilateral cervical lymphatic drainage plus resection of the inner third of the left clavicle plus disinsertion of the sternal hairpin and resection of the anterior wall of the sternal notch (Fig. 3). Finally, microsurgery was performed through reconstruction with an antero-lateral thigh (ALT) flap from the left thigh (Fig. 4).

Skin and soft tissue pathology, report a squamous cell infiltration of severe to moderately differentiated keratinizing cell carcinoma with invasion level hypodermis compromise.

No malignancy were report from soft tissues and the bones. No lymphovascular and neural invasion was not evident. Bilateral lymph node ganglionic groups III, IV, V were negative.

## 3 Discussion

Squamous cell carcinoma (SCC) is defined as the malignant proliferation of keratinizing cells of the epidermis. It is associated with chronic inflammation caused by radiation exposure (phototype I-II), chronic wounds (burn scars, ulcers, epidermolysis, fistulas), chemical carcinogens, immunosuppression, several genetic diseases, (that involve Bax immunoreactivity [psoriasis vulgaris]) and chronic infection, such as human papillomavirus infection [7,8]. SCC accounts for 15%–20% of nonmelanoma skin cancers, following basal cell carcinoma which accounts for 75%–80% [2,3]. The most common site for giant epithelial malignancies (basal cell carcinoma, SCC) is the scalp [5,9,10]; however, it has been known to appear on the face and ears.

In the present case, although the affected area was not one of the most frequent sites for this cancer, cervicothoracic region receives considerable solar exposure. Worldwide, head and neck cancers have the seventh highest mortality, of which 5.8% cases occur in Latin America [5,11]. In recent years, there has been an increase in the presentation of SCC in people older than 75 years [2]. The most frequent reasons for delayed diagnosis include low social status, poor personal hygiene, and fear of diagnosis and the possible consequences [12] as in our patient. SCC has more aggressive biological behavior when it is associated with risk factors such as smoking and alcohol consumption, which are observed in 75% of SCC cases.

Different histopathological types are observed in SCC. One is the verrucosa type, which is characterized by a well differentiated lesion showing locally invasive growth, with low metastatic potential and exophytic growth, as seen in our patient. Other variants include fusiform (which occurs more frequently in elderly patients), desmoplastic (highly infiltrative growth), acantholytic, and adenosquamous [13]. Its diagnosis is clinical, histological and for the stratification of these lesions, it must be supported by the use of high sensitivity imaging aids, such as CT and / or Magnetic Resonance Imaging (MRI) with contrast, as performed in this case.

Generally, early stage cancers show favorable clinical response and prognosis; however, a small proportion of SCCs present with rapid and aggressive growth with significant infiltration of perilesional tissues, eventually growing into a giant SCC (size > 5 cm). This is common in patients with particular sociodemographic characteristics, such as low educational level, poor personal hygiene, difficulty in accessing health services, and fear of possible cancer management [14], as observed in the present case. Some studies have even reported a relationship between psychiatric disorders and this type of disease [10,14].

Additionally, it is not uncommon for patients to choose home treatments for extended periods of time, as reported by a descriptive study in which 84.3% of the sample population used alternative therapies for an average of 11 months [10]. Our patient did not mention using cures or "home remedies". It is important to recognize factors predicting poor prognosis in this disease; following are some such factors: size of lesion > 2 cm, location on the ears and lips, area of no sun exposure, injuries secondary to radiation, previous wounds, burns, chronic inflammatory conditions, infiltration > 6 mm, positive borders, immunocompromised status, and recurrent injuries. These factors predict the risk of tumor recurrence and survival. Currently, the goal of giant SCC management is the achievement of better overall survival rates [12].

SCC treatment depends on location of the lesion, involvement of neighboring structures, functional level of the patient, and the patient’s acceptance of the proposed management strategy. Surgical removal with intraoperative margins > 2 cm still remains the fundamental strategy, with a cure rate of 95% with negative margins [15].

For tumors smaller than 2 cm, a margin greater than 5 mm is maintained, and for tumors greater than 2 cm in size and 6 mm in depth, a margin greater than 1 cm must be maintained.

The indications of sentinel lymph node biopsy, are high risk patients, stage III (TNM) classification for cutaneous carcinoma of the head and neck, perineural invasion, and Breslow 4 (between 2–4 mm) [13]. Our patient did not fulfill any of the mentioned criteria, for that reason were not performed the procedure.

In a meta-analysis of 19 reports, 130 non-anogenital SCC patients were found to have positive sentinel lymph nodes in 12.3% of patients had tumors > 2 cm in diameter, 11% had T2 tumors, and 60% had T4 tumors.

If the initial resection is incomplete, the recurrence rate increases to 75% and 95% 2 and 5 years after initial diagnosis, respectively. Metastasis occurs mainly to the regional lymph nodes, followed by the lung, and liver; it rarely occurs in the brain, skin, and bones [13].

Radiotherapy is used as neoadjuvant treatment in cases of involving large tumors, special localization as face or hands, and as adjuvant management is useful in cases of R0 patients, perineural compromise, positive margins, impossibility of total surgical resection, tumors in head and neck, trunk, and limbs undergoing lymph node dissection.

Radiotherapy is also used as a palliative treatment depending on comorbidities and inoperable critireas, such as contraindicated multiple tumors, warty type, genodermatosis (xeroderma, cellular nevus), scleroderma, tumors due to irradiation, poorly vascularized tumors, advanced injuries, joints and tendons.

Other alternative therapies, such as cryosurgery and electrochemotherapy, are also available [10]. The benefit of stage IV chemotherapy against metastatic disease has been reported; however, it is related to high hepatorenal toxicity in elderly patients. Currently, insufficient evidence is available for biological therapy; however, the use of monoclonal antibodies (cetuximab, panitumumab) or small molecule kinase inhibitors (erlotinib, gefitinib, and dasatinib) has been described against metastatic carcinoembryonic antigen (CEA) tumors.

According to the European consensus of 2016, carried out by the European Dermatology Forum (EDF), the European Association of Dermato-oncology (EADO) and the European Organization of Research and Treatment of Cancer (EORTC), clinical examinations must be performed annually for 5 years for patients with low risk; in patients with high risk, clinical examinations should be performed every 4 months during the first two years, every 6 months from the third to the fifth year, and then annually.

Ultrasound control should be performed in the clinical follow-up of patients for two years, and in cases of advanced or regional disease every 3 months for 5 years, and CT and/or MRI should be performed every 6 months for 5 years as there is a high risk of regional relapse and distant metastasis, with a high probability of occurrence of new skin tumors [12].

## 4 Conclusion

SCC of the skin is an aggressive and rapidly growing malignant tumor that can reach an enormous size if neglected. Early detection can confer good patient prognosis, reducing the risk of morbidity and mortality. The invasiveness of the tumor depends on its size, anatomical location, and histological subtype. Multidisciplinary management, such as the type used in this case, allows for a better prognosis. Surgery is the mainstay of treatment even for giant tumors, followed by oncological monitoring for reducing the risk of metastatic spread.

## Sources of funding

Nothing to declare.

## Ethical approval

The study is exempt from ethnical approval in our institution.

## Consent

Written informed consent was obtained from the patient for publication of this case report.

## Author contribution

Dr. Lopez, Dr Fory and Dr Conrado: Evaluation and post-operative managementof the case along with surgical assistance.

Dr. K. Moreno, Dr Garcia, Dr Buitrago and Dr Mogollon: Performed the surgical technique.

Dr. Pedraza, Dr Lopez: Assisted the surgical procedure.

## Registration of research studies

N/A.

## Guarantor

Mauricio Pedraza Ciro

## Provenance and peer review

Not commissioned, externally peer-reviewed.

## Declaration of Competing Interest

Nothing to declare.
